# Effect of Nanoencapsulated Alginate-Synbiotic on Gut Microflora Balance, Immunity, and Growth Performance of Growing Rabbits

**DOI:** 10.3390/polym13234191

**Published:** 2021-11-30

**Authors:** Nesrein M. Hashem, Nourhan S. Hosny, Nagwa I. El-Desoky, Mohamed G. Shehata

**Affiliations:** 1 Department of Animal and Fish Production, Faculty of Agriculture, Alexandria University, Alexandria 21545, Egypt; enagwa278@gmail.com; 2 Department of Livestock Research, Arid Lands Cultivation Research Institute, City of Scientific Research and Technological Applications (SRTA-City), New Borg El Arab, Alexandria 21934, Egypt; nourhansaad80@yahoo.com; 3 Department of Food Technology, Arid Lands Cultivation Research Institute, City of Scientific Research and Technological Applications (SRTA-City), New Borg El Arab, Alexandria 21934, Egypt; gamalsng@gmail.com

**Keywords:** alginate, synbiotic, moringa, yeast, gut microflora, immunity, antioxidant

## Abstract

A synbiotic comprising *Saccharomyces cerevisiae* yeast (SCY) and *Moringa oleifera* leaf extract (MOLE) has been encapsulated using nanotechnology. This duo is used as a dietary supplement for growing rabbits. Physicochemical analyses, in vitro antimicrobial activity, and gastrointestinal system evaluation were used to evaluate the quality of the nanofabricated synbiotic. The in vivo study was conducted using 40-day-old male growing rabbits (n = 16 rabbits/group) to evaluate the effect of the nanofabricated synbiotic on the health and growth performance of examined rabbits. Rabbits were equally allocated into four groups; (a) NCS, which received a basal diet supplemented with a noncapsulated 11 × 10^12^ CFU SCY + 0.15 g MOLE/kg diet, (b) LCS: those receiving a nanoencapsulated 5.5 × 10^12^ CFU SCY + 0.075 g MOLE/kg diet, (c) HCS: those receiving an 11 × 10^12^ CFU SCY + 0.15 g MOLE/kg diet, and (d) CON: those receiving a basal diet without treatment (control). The treatments continued from day 40 to day 89 of age. During the experimental period, growth performance variables, including body weight (BW), feed consumption, BW gain, and feed conversion ratio were recorded weekly. Blood samples were collected on day 40 of age and immediately before the start of the treatments to confirm the homogeneity of rabbits among groups. On day 89 of age, blood samples, intestinal, and cecal samples were individually collected from eight randomly selected rabbits. The size and polydispersity index of the nanofabricated synbiotic were 51.38 nm and 0.177, respectively. Results revealed that the encapsulation process significantly improved yeast survival through the gastrointestinal tract, specifically in stomach acidic conditions, and significantly increased in vitro inhibitory activities against tested pathogens. Furthermore, treatments had no negative effects on hematobiochemical variables but significantly improved levels of blood plasma, total protein, and insulin-like growth factor-l. Compared to the CON, NCS, and LCS treatments, the HCS treatment increased the amount of intestinal and cecal yeast cells (*p* < 0.05) and *Lactobacillus* bacteria (*p* < 0.05) and decreased number of *Salmonella* (*p* < 0.05) and *Coliform* (*p* = 0.08) bacteria. Likewise, both LCS and HCS significantly improved the small intestine and cecum lengths compared to CON and NCS. The HCS treatment also significantly improved BW gain and feed conversion compared to CON treatment, whereas the NCS and LCS treatments showed intermediate values. Conclusively, the nanoencapsulation process improved the biological efficiency of the innovative synbiotic used in this study. A high dose of encapsulated synbiotic balanced the gut microflora, resulting in the growth of rabbits during the fattening period.

## 1. Introduction

The profitability of rabbit farms depends on the efficacy of weanling rabbits to grow efficiently and cope with harsh environmental conditions during their fattening period. Therefore, during the production cycle, farm animals like rabbits are exposed to many management and environmental stresses that cause imbalances in their intestinal ecosystem and immunity. These imbalances make the animals more sensitive to pathogenic infections derived from many digestive disorders, such as diarrhea, bloat, and acidosis, particularly in growing animals [1]. Accordingly, enhancing their gastrointestinal microbiota ecosystem is proposed as a suitable solution for tackling previous disorders. Commonly, antibiotics are used to control pathogens and protect the health of the gut [2]. However, long-term use and misuse of antibiotics has led to the manifestation of multidrug antimicrobial resistance, which threatens both human and animal health. These health-related hazards, therefore, triggered the European Union Commission to ban the use of antibiotics as growth promoters in animal diets in 2003 ([3], Regulation 1831/2003/EC on additives for use in animal nutrition, replacing Directive 70/524/EEC on additives in feeding-stuffs). This perplexing situation between the forbidden use of antibiotics and the need to find safe antibiotic alternatives has forced researchers to explore the biological role of some natural feed additives with antimicrobial activities, such as probiotics, prebiotics, organic acids, and herbal extracts [4,5]. Among these feed additives, prebiotics, probiotics, and synbiotics are effective tools. Probiotics, such as *Lactobacillus* spp., *Streptococcus* spp., *Bacillus* spp., and *Saccharomyces* spp., are live microorganisms that can be used as direct-fed microbial feed supplements to sustain gastrointestinal microflora eubiosis [6]. They can resist enteric diseases caused by enteric pathogens, such as *Escherichia coli* and *Clostridium perfringens* [7]. Additionally, prebiotics (nutrients for the intestinal microbiota; soluble fibers, polyphenols, and polyunsaturated fatty acids) can support gastrointestinal microflora eubiosis, mainly through replenishment of beneficial microflora. Therefore, adding probiotics and prebiotics can enhance growth performance, decrease digestive disorders in growing animals, and reduce medication costs during the production cycle [8,9]. The possibility of getting the benefits of both probiotics and prebiotics can also be achieved through synbiotics. Synbiotics are a mixture of probiotics and prebiotics that now considered important tools for the maintenance of animal health. Furthermore, they can improve appetite, feed digestion and efficiency, immune functions, oxidative status, and yield and quality of meat and milk when they are included in animal diets [10,11]. Synbiotics mainly act by improving the number of beneficial bacteria and reducing the pathogen load in the gastrointestinal tract of farm animals. Therefore, it has been established that including synbiotics in feed is safe, ecofriendly, and reduces the demand for antibiotic-based growth promoters [10]. In this respect, finding the proper combinations of probiotics and prebiotics that cause significant improvements in animal productivity remains the major challenge in formulating potential synbiotics. Prebiotics used in synbiotic formulas are commonly sources of carbohydrates. However, recent studies have underlined the possibility of including polyphenols and fatty acids as prebiotics that not only aid in improving animal performance but also produce functional animal products. Furthermore, these originated plant materials possess antimicrobial activities, modulate cecal fermentation, and improve short-chain fatty-acid production, thereby influencing total animal growth [8,12]. Thus, we expect that using phenolic-rich plants as prebiotics in the synbiotic formula would provide additional functions to the product, as phenolic compounds themselves can improve immune functions [1], modulate cecal fermentation [8,9], and improve blood metabolites, as well antioxidant activity. *Moringa oleifera* is one of the active component-rich plants that can be typically used as a prebiotic. This plant has an impressive range of polyphenols, amino acids, fatty acids, vitamins, and minerals that can maintain gut microflora eubiosis. The addition of *Moringa oleifera* leaf extract can increase the growth of gut probiotic bacteria, such as lactic-acid bacteria [13].

In terms of probiotics, yeast (*Saccharomyces cerevisiae*) and Gram-positive bacteria (*Bacillus*, *Enterococcus*, *Lactobacillus*, *Bifidobacterium*, *Pediococcus*, and *Streptococcus*) are the common probiotics used in the preparation of direct microbial feed additives [14]. Nevertheless, besides finding effective probiotic and prebiotic combinations, achieving adequate efficiency of synbiotic products depends on maintaining probiotic survival ability and prebiotic stability against processing, storage, and gastrointestinal conditions. Therefore, developing encapsulation techniques facilitates the protection, as well as the controlled and targeted release, of bioactive molecules [15], which can be used to prepare a more efficient synbiotic for farm animals. Therefore, this study tested the efficiency of a newly innovated synbiotic feed additive comprising moringa leaf extract as a prebiotic and *Saccharomyces cerevisiae* yeast as a probiotic using nanoencapsulation technology. Then, we examined their effects on the growth performance and health of growing rabbits.

## 2. Materials and Methods

### 2.1. Fabrication of Encapsulated Synbiotic

A weight of 250 g fine powder of *Moringa oleifera* leaf was extracted using 1000 mL of ethanol solution (70% *v*/*v*) at 40 °C for 72 h [12]. The *Moringa oleifera* leaf extract (MOLE) was then lyophilized and used to synthesize a sodium alginate nanocomplex by adopting the ionic-gelation method. Under gentle magnetic stirring, the MOLE (1.5% *w*/*v*), TWEEN 80 (0.5% *v*/*v*), and SCY (10^12^ cell/colony-forming units (CFU)) were mixed with 100 mL of sodium alginate solution (1% *w*/*v*), after which the solution was added dropwise using a syringe into 50 mL of calcium chloride (CaCl_2_; 0.5% *w*/*v*) under gentle magnetic stirring [16]. Subsequently, the formed beads were separated, washed three times with distilled water, lyophilized, and then stored at 5 °C until later use [17].

### 2.2. Physicochemical Properties and Encapsulation Efficiency

Particle size and size distribution (Polydispersity index, PdI) of the free alginate-CaCl_2_ nanoparticles and encapsulated synbiotic (Moringa and yeast cells) were measured using a Zetasizer instrument (Malvern Instruments, Malvern, UK), which is based on dynamic light-scattering techniques [18]. The encapsulation efficiency (EE, %) was also determined by estimating the encapsulation efficiency of phenolic compounds in MOLE as an indicator of prebiotic encapsulation and that of SCY cells as an indicator of probiotic encapsulation [19]. The encapsulation efficiency of alginate-CaCl_2_ nanoparticles for MOLE and SCY was calculated using the following equation: EE = (concentration of phenolic compounds or number of SCY cells used during encapsulation—concentration of free phenolic compounds or number of SCY cells in supernatant) × 100/concentration of phenolic compounds or number of SCY cells used during encapsulation.

### 2.3. In Vitro Gastrointestinal Simulation Tests

Simulated oral (salivary), gastric, and intestinal fluids were prepared according to the method proposed by Minekus et al. [20] to test the viability of nonencapsulated and encapsulated yeast cells through the gastrointestinal tract. At the oral phase, five grams of capsule beads was mixed with 7 ml of simulated salivary fluid electrolyte stock solution. Then, 0.5 mL of salivary α-amylase solution (1500 units/mL) was added. Next, 50 µL of 0.3 M CaCl_2_ and 2 mL of water was added, and the solution was thoroughly mixed. The pH was adjusted to 7.0, and the oral bolus thus prepared was kept at 37 °C for 2 min. At the gastric phase, the obtained oral bolus was mixed with 7.5 mL of simulated gastric fluid electrolyte stock solution. Then, 1.6 mL of porcine pepsin stock solution (25,000 units/mL) prepared with simulated gastric fluid electrolyte stock solution was thoroughly mixed with the bolus, and 5 µL of 0.3 M CaCl_2_ and 0.69 µL of water was added. Then, the pH was adjusted to 3.0 with HCl (0.1 N). The gastric chime thus prepared was kept in a shaking incubator at 100 rpm at 37 °C for 2 h. At the intestinal phase, the obtained gastric chime was mixed with 11 mL of simulated intestinal fluid electrolyte stock solution, 5.0 mL of a pancreatin solution (800 units/mL) prepared with simulated intestinal fluid electrolyte stock solution, 2.5 mL of fresh bile (160 mM in fresh bile), 40 µL of 0.3 M CaCl_2_, and 1.5 mL of water. The pH of the mixture was adjusted to 7.0 using NaOH. The intestinal phase thus prepared was kept in a shaking incubator at 100 rpm at 37 °C for 3 h. Following the end of each stage, the number of live yeast cells was counted, and the survivability of SCY cells was calculated [19].

### 2.4. In Vitro Antimicrobial Activities of Synbiotics

The agar-well diffusion method was used to determine the diameters of inhibition zones between the nonencapsulated and two levels of the nanoencapsulated synbiotic against five pathogenic strains, including *Escherichia coli* (BA 12296B), *Staphylococcus aureus* (NCTC 10788), *Candida albicans* (ATCC MYA-2876), *Listeria monocytogenes* (ATCC 19116), and *Salmonella senftenberg* (ATCC 8400). Tests were performed in triplicate, and the results were presented as the mean ± standard error [21].

### 2.5. Animal Husbandry and Experimental Design

This part of the study was conducted at the Laboratory of Rabbit Physiology Research, Agricultural Experimental Station, Alexandria University, Egypt. The study was conducted according to the guidelines of the Declaration of Pharmaceutical and Fermentation Industries Development Center. The protocol and procedures were approved by the Institutional Animal Care and Use Committee, SRTA-city (registration number #311Z0521).

Under similar management and hygiene conditions, growing, V-line, male rabbits (40 days old), weighing 847 ± 25.87 g at allocation (initial weight), were individually kept in standard galvanized wire cages (40 cm × 50 cm × 35 cm) equipped with automatic drinkers and feeders. The mean of indoor temperature, relative humidity, and daylight length during the experimental period (March and April) was 23.14 ± 1.27 °C, 52.47 ± 9.15%, and 11.78 ± 0.74 h, respectively.

Rabbits were fed on a pellet diet with quantities covering their daily nutritional requirements [22]. The diet comprised (g per each kg diet): 280 alfalfa hay, 250 wheat bran, 180 barley, 180 soybean meal, 60 yellow corn, 30 molasses, 10 CaCO_3_, and 10 NaCl, which offers 17.90% crude protein and 10.05 MJ/kg digestible energy. Rabbits were equally allocated into four groups (n = 16/group) and received synbiotic product as follows: noncapsulated 11 × 10^12^ SCY CFU + 0.15 g MOLE/kg diet (NCS), encapsulated 5.5 × 10^12^ SCY CFU + 0.075 g MOLE/kg diet (LCS), encapsulated 11 × 10^12^ SCY CFU + 0.15 g MOLE/kg diet (HCS), or no treatment diet (control, CON). Each diet was administered for 50 days (from day 40 to day 89 of age).

### 2.6. Blood Plasma, Biochemical Attributes, Antioxidant Indicators, and Immunological Variables

For blood biochemical attributes, blood samples were collected on days 40 and 89 of age from eight randomly chosen rabbits in each experimental group. Blood samples were harvested from the marginal ear vein of each rabbit using heparinized tubes, after which concentrations of hemoglobin were assessed colorimetrically using commercial kits (Biosystems S.A., Costa Brava, Barcelona, Spain). Numbers of erythrocytes and leukocytes and their types were determined [23]. Next, blood plasma metabolites, including total protein, albumin, and glucose, were analyzed using commercial kits (Biodiagnostic, Giza, Egypt). The linearity of the methods was up to 10.0 g/dL, 7.0 g/dL, and 500 mg/dL for total protein, albumin, and glucose contents, respectively. The values of blood plasma globulin were obtained by subtracting those of the albumin from the corresponding values of the total protein. The total antioxidant capacity and malondialdehyde concentrations in the plasma were also determined as indicators of the redox status of the plasma content (Biodiagnostics, Giza, Egypt). The linearity of the methods was up to 2 mM/L and 100 nmol/mL, respectively. Enzyme-linked immunosorbent assay was applied to assess concentrations of immunoglobulin-G, immunoglobulin E, and immunoglobulin A (IBL America Immuno-Biological Laboratories, Inc., Minneapolis, MN, USA). The sensitivity and specificity of the assays exceeded 96%. Interleukins-1 (IL-1) concentrations were also determined using commercial kits, with the minimum detectable concentration being 500 pg/mL (IBL America Immuno-Biological Laboratories, Inc., Minneapolis, MN, USA). An immunoassay kit obtained from Quantikine IGF-l Immunoassay, R&D Systems, Minneapolis, MN, USA was used to measure insulin-like growth factor l (IGF-l) concentrations. The mean minimal detectable concentration of IGF-l was 0.026 ng/mL. Additionally, the mean of the intra- and inter-assay CVs was 3.0% and 8.0%, respectively.

### 2.7. Intestinal and Cecal Microbial Count and Fecal Score Evaluation

On day 89 of age, 32 rabbits, previously used for blood sampling, were sacrificed [8]. The small intestine and cecum were then separated, after which the length of each part was measured. The intestine and cecum were also ligated using light twine before separating the cecum from the small intestine. Subsequently, the first part of the small intestinal tract and the last part of the cecum were removed and stored in sterile bags at −4 °C. For bacterial enumeration, the intestinal and cecal contents were separately diluted using sterile ice-cold anoxic PBS and homogenized for 3 min in a stomacher. In the next step, each homogenate was serially diluted from 10^−1^ to 10^−7^. Dilutions were then plated in duplicate on selective agar media for the identification of target bacterial groups, and the enumeration results were expressed as CFU log 10/g. In particular, the Sabouraud Dextrose agar was used for yeast counts; de Man, Rogosa, and Sharpe agar for *Lactobacillus* counts; the MacConkey agar media for *coliform* counts; and *Salmonella* and *Shigella* agar plates for *Salmonella* counts. Plates were then incubated at 37 °C from 24 h to 72 h [24,25]. Fecal scores were assigned from 1 to 4, where score 1: normal; 2: soft; 3: mixed soft and liquid; and 4: completely liquid [26].

### 2.8. Growth Performance

BW and the wight of feed residues were recorded weekly during the entire experimental period using a relevance table scale (Honder Weighing Scale Co., Ltd., Taipei City, Taiwan). Data were then used to estimate feed intake, BW gain, and feed conversion ratio [1]. 

**Figure 1:**
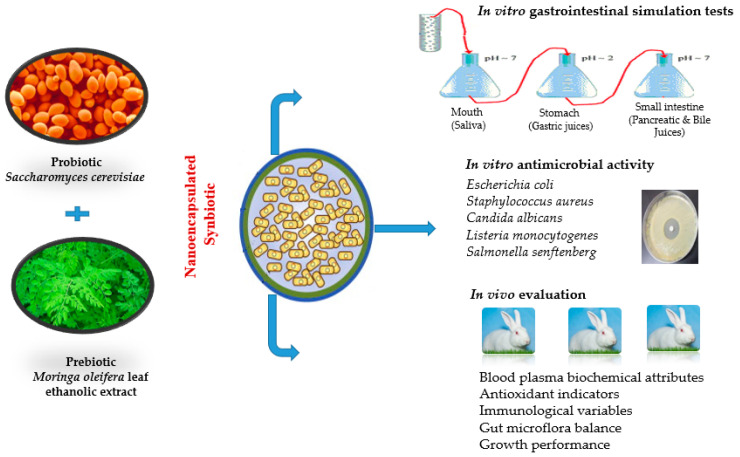
A flowchart for the in vitro and in vivo tests performed in this study to evaluate the biological activity of the fabricated nanoencapsulated synbiotic.

A flowchart for the in vitro and in vivo studies is shown in Figure 1.

### 2.9. Statistical Analysis

The statistical analysis system (SAS, Version 8. Cary, NC, USA: SAS Institute; 2001) software was used for analyzing all the results. Hematological, immunological, and biochemical parameters were also analyzed using the generalized linear model. Furthermore, the differences between treatment means were tested using Tukey’s range test. All results were shown as mean ± standard error, and statistical significance was set at *p* < 0.05.

## 3. Results

### 3.1. Physicochemical Properties and Encapsulation Efficiency

Physicochemical characteristics and the encapsulation efficiency of alginate-CaCl_2_ nanoparticles for MOLE and SCY are shown in Table 1. The encapsulation efficiency of MOLE (Phenolic compounds) and SCY by alginate-CaCl_2_ was 57.55% and 71.17%, respectively. The average size and PdI of alginate-CaCl_2_ nanoparticles, including alginate-CaCl_2_ encapsulated synbiotic nanoparticles were 195.10 nm and 0.457 versus 51.38 nm and 0.177, respectively.

### 3.2. In Vitro Gastrointestinal Simulation Tests and the Antimicrobial Activity Results of Administered Synbiotics

In vitro gastrointestinal simulation tests revealed that the highest (*p* < 0.05) protective effect for the survivability of SCY on gastric and intestinal enzymatic digestion was observed in the HCS treatment group, followed by the LCS group, whereas lower protective effect was observed in the NCS group (Table 2).

The antimicrobial activity test of administered synbiotics revealed that HCS treatment resulted in the highest (*p* < 0.05) inhibitory effect on tested pathogenic bacteria and fungi, including *Escherichia coli* (BA 12296), *Staphylococcus aureus* (NCTC 10788), *Salmonella* and *Listeria monocytogenes* (ATCC 19116), as well as *Candida albicans* (ATCCMYA-2876) (Table 3), followed by the LCS and NCS treatments.

### 3.3. Hemato-Biochemical and Immunological Responses

Results showed that treatments had no effect on all hematological variables (Table 4). The concentration of blood-plasma total protein was found to be higher (*p* < 0.05) in the HCS treatment than other treatments, as well as the control treatment. However, all synbiotic-based treatments increased blood-plasma albumin concentrations (*p* < 0.05) compared to the CON treatment (Table 5). Furthermore, treatments did not affect concentrations of blood-plasma globulin, glucose, and total antioxidant capacity (Table 5). Nevertheless, all synbiotic-based treatments significantly decreased concentrations of blood-plasma MDA compared to the CON treatment (Table 5). Different synbiotic additives did not affect levels of blood-plasma immunoglobulins G and A. However, the HCS treatment significantly decreased immunoglobulin E compared to the CON treatment. The HCS treatment also significantly increased insulin-like growth factor-l compared to the CON treatment, whereas NCS and LCS recorded intermediate values. Besides, treatments had no effects on interleukin-l (Table 6).

### 3.4. Intestinal and Cecal Microbial Count and Fecal Score Evaluation

Treatment with either nonencapsulated or both levels of nanoencapsulated diets significantly increased the number of beneficial intestinal and cecal microbes (yeast and lactic-acid bacteria) at day 89 of age, and the greatest (*p* < 0.05) positive effects were observed with HCS treatments compared to control and nonencapsulated treatment (Table 7). However, an opposite trend was observed in the effects of treatments on the numbers of pathogenic microbes (*Coliform* spp. and *Sallmonella* spp.) (Table 7). Treatment with both levels of nanoencapsulated synbiotic also improved (*p* < 0.05) the length of small intestine and cecum and decreased (*p* < 0.05) the values of fecal scores compared to control and nonencapsulated treatments (Table 7).

### 3.5. Growth Performance

Compared to the CON treatment, the HCS treatment resulted in higher BW on day 89 (final body weight) and overall BW gain, whereas both NCS and LCS treatments resulted in intermediate values. Furthermore, all synbiotic treatments did not affect overall feed intake compared to the CON treatment. However, both NCS and HSC treatments significantly increased overall feed intake compared to the LCS treatment. Results also showed that the HCS treatment group significantly decreased their overall feed conversion ratio compared to the CON treatment, whereas both NCS and LCS treatments resulted in intermediate values (Table 8).

## 4. Discussion

In 2004, Gibson and Roberfroid introduced the term “synbiotic” as “a mixture of probiotics and prebiotics that beneficially affects the host by improving the survival and implantation of live microbial dietary supplements on the gastrointestinal tract, selectively stimulating growth and activating the metabolism of one or a limited number of health-promoting bacteria, thereby improving host welfare.” Therefore, in this study, SCY (probiotic) and MOLE (prebiotic) were used to develop a synbiotic product. The selection of these two materials was based on promising biological activities previously reported in several studies [1,8]. Thus, *S. cerevisiae* is one of the probiotic microbial species that secret many extracellular metabolites, such as vitamins, enzymes, peptides, alcohols, esters, and organic acids [27], which have several biotherapeutic effects [28]. These metabolites can then positively affect absorption and use of feed, as well as improve gut microflora eubiosis, immunity, and growth performance via different mechanisms [29]. Alternatively, Moringa (*Moringa oleifera* Lam), a tropical/subtropical tree, encompasses many naturally occurring nutrients and active components, such as simple sugars, amino acids, fatty acids, vitamins, phenols, and cytokinin-type hormones [8,30]. Therefore, this plant displays antioxidant, anti-inflammatory, antipyretic, antiulcer, diuretic, antihypertensive, cholesterol-lowering, hepatoprotective, antibacterial, and antifungal activities [31].

This study is the first attempt at investigating the effects of feeding rabbits with a synbiotic product fabricated using nanotechnology approaches. In this study, we used the nanoencapsulation ionic-gelation method to develop a new feed additive that encompasses MOLE and SCY (synbiotic formula) as a potential attempt to improve growth performance and health of growing rabbits using natural feed additives. Furthermore, we used this technology to overcome some obstacles proposed to restrict the efficiency of probiotics or prebiotics in animal feeding, as many probiotic microbial species, particularly yeast, cannot efficiently cope with the biological conditions of the gastrointestinal tract [32,33]. Similarly, many prebiotic-active components with desirable biological effects are highly unstable and susceptible to oxidation. Hence, encapsulation technology offers the advent of improving stability, increasing bioavailability, and sustaining release of both probiotic and prebiotic-based formulas [34]. Encapsulation is a process used to entrap active agents within a carrier material, and it is a useful tool to improve the viability of living cells, including the active phytochemicals therein, during the inclusion of these cells in feeds in biological systems, such as the gastrointestinal tract. Additionally, encapsulation can promote the controlled release of bioactive compounds and optimize delivery to the site of action, thereby potentiating the efficacy of the respective probiotic strains [32]. Therefore, according to our results, alginate-CaCl_2_ nanoparticles encapsulated more than 50% of the MOLE and SCY present (synbiotic formula). These findings support the relevance of the technique used in this study to yield synbiotic nanoparticles with physicochemical characteristics suitable for maintaining the sufficient efficiency needed under gastrointestinal-tract conditions. Furthermore, the ionic-gelation method used is an efficient and low-cost encapsulation technique that does not require specialized equipment, high temperature, or organic solvents, thereby making it suitable for both hydrophobic and hydrophilic compounds [35,36]. These results are in agreement with those reported by Arriola et al. [36], who reported on the importance of encapsulation techniques in maintaining the viability of probiotic microbial species.

This study also reported on the biological activity of prebiotic-active components, such as polyphenols. In rabbit farms, microbial and fungal pathogens can trigger serious health problems and economic losses. For example, staphylococcal infections cause substantial economic losses in commercial farms, with clinical signs in more than 60% of rabbitries. Hence, it has been reported that the infection of rabbits with *S. aureus* is associated with suppurative dermatitis, abscesses, pododermatitis, and mastitis, including chronic mastitis, which accounts for the culling of diseased animals in rabbitries [37]. Furthermore, *Listeria monocytogenes* have the ability to cross the intestinal, blood–brain, and fetoplacental barriers [38]. Studies have also shown that *Candidiasis* can affect the skin integrity and mucosal membranes of the gastrointestinal tract [39]. Nevertheless, in this study, synbiotics, either in nonencapsulated or nanoencapsulated forms, showed strong in vitro antimicrobial activities against some pathogenic microbes, such as *Escherichia coli* (BA 12296), *Staphylococcus aureus* (NCTC 10788), *Listeria monocytogenes* (ATCC 19116), and *Candida albicans* (ATCC MYA-2876). These effects were attributed to the multi-antimicrobial activities of innovative synbiotic components. For example, MOLE can inhibit both Gram (+) and Gram (−) bacteria [40]. This observed activity is also related to the presence of phenolic acids (gallic acid, chlorogenic acid, ferulic acid, and ellagic acid) and flavonoids (quercetin and kaemferol) present in MOLE [41,42]. Additionally, in this study, the potential of innovative synbiotics to maintain the gastrointestinal tract eubiosis was confirmed, which is compatible with in vitro results. These results are also in agreement with those of Shanmuganathan et al. [43], who reported that the inclusion of SCY in rabbit diets resulted in higher numbers of intestinal and cecal *Lactobacilli* and yeast compared to a control diet. A similar potential of the particular synbiotic used in that study also modulated the composition of the cecal microflora and potentially suppressed pathogenic bacteria, such as coliforms and *Salmonella* [44,45]. Furthermore, a study reported that continual probiotic supplementations of animals’ feed enhanced the proliferation of beneficial intestinal microflora through two routes: first, by competitive insularity, and second, through antagonistic activities toward pathogenic bacteria [26]. In this way, probiotics can leverage the intestinal microbiota and the host’s health, thereby increasing nutrient use, producing antimicrobial compounds, and stimulating the immune system [46]. The bactericidal effect of probiotics was also proposed to be due to the production of different antimicrobial compounds by probiotic strains, such as organic acids, diacetyl, hydrogen peroxide, and carbon peroxide [26]. Hence, given our innovative synbiotic constitutes, SCY decreased the number of undesirable bacteria, which is proposed to be through competition for sites during pathogens colonization [47]. Additionally, Hashem et al. [8] also confirmed the prebiotic properties of MOLE and its enrichment with nutrients that support the proliferation of beneficial gut microflora. Therefore, alkaloids and organ-sulfur compounds, such as S-phenylmercapturic acid, are abundant in MOLE. Such compounds have strongly selective bactericidal activities against pathogenic bacteria, including *Escherichia coli*, in addition to their anti-inflammatory and immunomodulatory activities [8]. Furthermore, in this study, the synbiotic formulation did not show negative impacts on heamato-biochemical parameters, thus indicating its safety. The hemolytic activity (normal number of erythrocytes and levels of hemoglobin), including those allergic responses (decreased immunoglobulin E levels) that can be observed following treatment with some prebiotics or prebiotics, was not seen in this study either. Moreover, the synbiotic formula improved protein content, specifically albumin and redox status. Blood-plasma albumin is the most abundant blood protein in mammals, and the liver produces this protein. Therefore, the content of albumin reflects the efficiency of protein digestion and usage processes. Biologically, one of the most important functions of albumin is the regulation of the osmotic pressure of blood. Thus, its deficiency results in chronic hepatic or gastrointestinal diseases [48,49].

## 5. Conclusions

The encapsulation process using alginate-CaCl_2_ nanoparticles as cargo improved the biological efficiency of an innovative synbiotic, compositing *Moringa oleifera and Saccharomyces* *cerevisiae* by improving synbiotic stability against destructive gastrointestinal conditions. A high dose of encapsulated synbiotic also adjusted gut microflora constituents and enhanced immunity, including growth performance of rabbits during the fattening period. These positive effects on immunity and growth performance were related to the prevalence of beneficial intestinal and cecal microorganisms, indicating the opportunity of using synbiotic formula, specifically in the nanoencapsulated form. These findings offer novel opportunities for more sustainable animal production, ensuring the maintenance of adequate animal health using natural feed additives.

## Figures and Tables

**Table 1:** Particle size and polydispersity index of free alginate-CaCl_2_ nanoparticles (free NP) and alginate-CaCl_2_ nanoparticles loaded with moringa extract and yeast cells (loaded NP) and encapsulation efficiency.

Item	Free NP	Loaded NP
Size (nm)	195.10	51.38
Polydispersity index	0.457	0.177
Encapsulation efficiency of Phenolic compounds, %	-	57.55
Encapsulation efficiency of Yeast cells, %	-	71.17

**Table 2:** Survivability of *Saccharomyces cerevisiae* cells in nonencapsulated (NCS) or two levels of nanoencapsulated synbiotic (LCS = low level and HCS = high level) during in vitro gastrointestinal simulation test.

Number of Live Cells, log CFU/mL	Treatment		
	NCS	LCS	HCS
Oral phase	12.3 ± 0.90 ^a^	12.60 ± 0.55	12.23 ± 0.87
Gastric phase	6.93 ± 1.11 ^b^	8.63 ± 1.02 ^ab^	10.40 ± 0.95 ^a^
Intestinal phase	4.70 ± 0.81 ^b^	6.23 ± 1.10 ^b^	8.70 ± 0.70 ^a^

^a,b^ Mean values in the same row followed by uncommon superscript letters are significantly different (*p* < 0.05).

**Table 3:** In vitro antimicrobial activities expressed as inhibition zone (mm) of the nonencapsulated synbiotic (NCS) or two levels of nanoencapsulated synbiotic (LCS = low level and HCS = high level).

Pathogenic Microorganism (Inhibition Zone, mm)	Treatment		
	NCS	LCS	HCS
*Escherichia coli* BA 12296	13.00 ± 1.32 ^c^	18.00 ± 1.00 ^b^	26.33 ± 1.04 ^a^
*Listeria monocytogenes* ATCC 19116	10.00 ± 0.86 ^c^	18.00 ± 1.00 ^b^	30.00 ± 1.73 ^a^
*Staphylococcus aureus* NCTC 10788	11.03 ± 1.26 ^c^	15.30 ± 1.08 ^b^	20.00 ± 2.09 ^a^
*Salmonella senftenberg* ATCC 8400	18.16 ± 0.76	0.00	0.00
*Candida albicans* ATCC MYA-2876	10.00 ± 2.00 ^c^	15.10 ± 0.95 ^b^	30.50 ± 2.17 ^a^

^a,b,c^ Means in the same row followed by uncommon superscript letters are significantly different (*p* < 0.05).

**Table 4:** Hematological attributes (red blood cells, RBC, and white blood cells, WBC, and their types) of rabbit supplemented with nonencapsulated synbiotic (NCS) or two levels of nanoencapsulated synbiotic (LCS = low level and HCS = high level) or no treatment (control, CON).

Treatment	Item						
	Lymphocytes, %	Monocytes, %	Eosinophil, %	Heterophil, %	WBC, × 10^3^/mL	RBC, × 10^6^/mL	Hemoglobulin, g/dL
At day 40 of age							
CON	39.90 ± 5.69	13.18 ± 2.06	12.49 ± 1.92	21.24 ± 0.01	29.17 ± 0.91	2.10 ± 0.08	10.16 ± 0.30
NCS	40.84 ± 2.24	13.48 ± 0.17	11.36 ± 0.06	21.25 ± 0.13	23.08 ± 0.05	2.09 ± 0.21	9.58 ± 0.31
LCS	38.27 ± 0.27	11.13 ± 0.24	11.26 ± 0.47	21.86 ± 0.57	24.34 ± 1.19	2.02 ± 0.36	10.08 ± 0.59
HCS	38.37 ± 0.50	12.94 ± 0.98	11.15 ± 0.69	20.61 ± 0.01	24.77 ± 1.53	1.69 ± 0.04	9.74 ± 0.59
*p*-value	0.418	0.472	0.762	0.318	0.356	0.729	0.860
At day 89 of age							
CON	42.01 ± 1.32	13.22 ± 1.20	11.74 ± 0.51	22.95 ± 0.18	25.97 ± 0.84	1.98 ± 0.32	10.92 ± 0.66
NCS	41.03 ± 1.38	14.04 ± 1.14	10.52 ± 0.69	21.47 ± 1.59	25.07 ± 0.88	1.67 ± 0.08	10.25 ± 1.54
LCS	43.08 ± 1.32	14.28 ± 1.25	12.96 ± 1.83	23.70 ± 0.43	24.01 ± 1.29	1.73 ± 0.13	11.68 ± 1.27
HCS	40.96 ± 0.62	12.98 ± 1.24	10.02 ± 0.88	21.99 ± 0.33	24.55 ± 1.53	1.86 ± 0.06	11.08 ± 0.97
*p*-value	0.581	0.827	0.306	0.316	0.690	0.632	0.853

**Table 5:** Blood-plasma biochemical constituents and oxidative status of rabbits supplemented with nonencapsulated synbiotic (NCS), two levels of nanoencapsulated synbiotic (LCS = low level and HCS = high level) or no treatment (control, CON).

Treatment	Item					
	Total Protein, g/dL	Albumin, g/dL	Globulin, g/dL	Glucose, mg/dL	Malondialdehyde, nmol/L	Total Antioxidant, Mm/L
At day 40 of age						
CON	6.25 ± 0.06	4.37 ± 0.32	1.88 ± 0.25	97.27 ± 0.92	5.93 ± 0.09	0.493 ± 0.57
NCS	5.57 ± 0.35	3.52 ± 0.3	2.04 ± 0.05	81.97 ± 1.23	4.78 ± 0.13	0.437 ± 5.53
LCS	5.97 ± 0.20	4.43 ± 0.22	1.55 ± 0.02	82.48 ± 0.27	5.53 ± 0.18	0.424 ± 1.76
HCS	4.98 ± 0.14	3.07 ± 0.27	1.91 ± 0.13	87.61 ± 1.16	5.69 ± 0.03	0.424 ± 1.97
*p*-value	0.393	0.202	0.457	0.416	0.379	0.508
At day 89 of age						
CON	5.21 ± 0.01 ^b^	3.37 ± 0.14 ^b^	1.84 ± 0.27	91.06 ± 1.09	5.02 ± 0.25 ^a^	0.446 ± 2.94
NCS	5.52 ± 0.27 ^b^	4.82 ± 0.18 ^a^	1.5 ± 0.21	93.73 ± 1.09	2.99 ± 0.17 ^b^	0.443 ± 9.99
LCS	6.03 ± 0.1 ^ab^	4.23 ± 0.34 ^a^	1.80 ± 0.29	93.05 ± 1.11	3.08 ± 0.08 ^b^	0.444 ± 8.49
HCS	6.43 ± 0.27 ^a^	4.02 ± 0.29 ^ab^	1.61 ± 0.54	94.72 ± 1.06	3.13 ± 0.32 ^b^	0.430 ± 1.76
*p*-value	0.045	0.023	0.888	0.186	0.005	0.360

^a,b^ Means in the same column followed by uncommon superscript letters are significantly different (*p* <0.05).

**Table 6:** Immunity and inflammatory (interleukin-l) indicators, including insulin-like growth factor-l of rabbits supplemented with nonencapsulated synbiotic (NCS), two levels of nanoencapsulated synbiotics (LCS = low level and HCS = high level), or no treatment (control, CON).

Treatment	Item				
	Immunoglobulin G, mg/dL	Immunoglobulin E, mg/dL	Immunoglobulin A, mg/dL	Interleukin-l, pg/mL	Insulin-like Growth Factor-l, ng/mL
At day 40 of age					
CON	1146.7 ± 6.38	7.99 ± 0.28	99.33 ± 1.42	20.99 ± 2.02	168.01 ± 0.36
NCS	963.1 ± 4.84	7.66 ± 0.52	87.92 ± 2.02	17.58 ± 1.98	158.92 ± 4.55
LCS	967 ± 1.97	7.39 ± 1.61	85.77 ± 0.56	20.79 ± 1.19	170.33 ± 5.79
HCS	955.4 ± 13.00	8.91 ± 0.72	83.46 ± 0.84	17.55 ± 0.60	155.74 ± 4.94
*p*-value	0.357	0.035	0.484	0.542	0.222
At day 89 of age					
CON	986.50 ± 3.84	11.44 ± 0.78 ^a^	95.31 ± 1.55	18.66 ± 0.13	163.05 ± 3.01 ^b^
NCS	965.58 ± 5.35	8.13 ± 1.71 ^ab^	89.30 ± 3.02	19.83 ± 2.33	159.46 ± 2.64 ^ab^
LCS	973.99 ± 13.38	9.79 ± 0.65 ^ab^	90.65 ± 4.73	18.97 ± 1.24	157.30 ± 5.42 ^ab^
HCS	983.34 ± 3.98	5.97 ± 0.65 ^b^	85.30 ± 2.35	19.33 ± 1.61	169.09 ± 2.49 ^a^
*p*-value	0.285	0.088	0.238	0.357	0.012

^a,b^ Means in the same column followed by uncommon superscript letters are significantly different (*p* < 0.05).

**Table 7:** Small-intestine microflora composition, length of small intestine and cecum, and fecal score of rabbits supplemented with nonencapsulated synbiotic (NCS), two levels of nanoencapsulated synbiotics (LCS = low level and HCS = high level), or no treatment (control, CON) at day 89 of age.

Item		Treatments			
		CON	NCS	LCS	HCS
Type of Microflora, log CFU/g					
Intestinal microflora					
Yeast		4.83 ± 0.65 ^d^	6.20 ± 0.20 ^c^	7.40 ± 0.72 ^b^	8.80 ± 0.3 ^a^
Lactic acid bacteria		6.53 ± 0.50 ^c^	7.30 ± 0.75 ^b^	8.16 ± 0.47 ^a, b^	8.53 ± 0.55 ^a^
*Coliform*		6.30 ± 0.70 ^a^	5.40 ± 0.45 ^ab^	5.13 ± 0.40 ^b^	3.20 ± 0.26 ^c^
*Salmonella*		5.96 ± 0.55 ^a^	4.90 ± 0.96 ^ab^	4.00 ± 0.10 ^cd^	3.46 ± 0.56 ^d^
Cecal microflora					
Yeast	3.06 ± 0.51 ^c^		4.87 ± 0.31 ^b^	5.73 ± 0.86 ^b^	7.03 ± 0.58 ^a^
Lactic acid bacteria	5.16 ± 0.35 ^d^		6.37 ± 0.64 ^c^	7.20 ± 0.52 ^b^	8.46 ± 0.32 ^a^
*Coliform*	8.13 ± 0.61 ^a^		7.10 ± 0.69 ^b^	6.00 ± 0.50 ^c^	5.16 ± 0.47 ^d^
*Salmonella*	7.63 ± 0.86 ^a^		6.63 ± 0.98 ^b^	4.96 ± 0.65 ^c^	3.83 ± 0.84 ^d^
Length of small intestine and cecum, and fecal score					
Small intestine length (Cm)	285 ± 30.36 ^b^		280 ± 10.15 ^b^	348 ± 17.58 ^a^	331.67 ± 21.69 ^a^
Cecum length (Cm)	95.00 ± 7.56 ^c^		107.07 ± 11.18 ^b^	112.67 ± 8.92 ^a^	114.50 ± 9.34 ^a^
Fecal score	1.15 ± 0.05 ^ab^		1.99 ± 0.03 ^a^	1.06 ± 0.04 ^b^	1.04 ± 0.02 ^b^

^a,b,c,d^ Means in the same row followed by uncommon superscript letters are significantly different (*p* < 0.05).

**Table 8:** Growth performance of rabbits supplemented with nonencapsulated synbiotic (NCS), two levels of nanoencapsulated synbiotics (LCS = low level and HCS = high level), or no treatment (control, CON).

Item	Age of Rabbits			
**Live BW, g**	BW40	BW54	BW68	BW89
CON	846 ± 25.87	1046.50 ± 29.07	1253.50 ± 65.09	1761.67 ± 49.26 ^b^
NCS	847 ± 28.87	1018 ± 28.10	1268.50 ± 48.93	1853.89 ± 42.02 ^ab^
LCS	848 ± 0.50	1028.50 ± 48.45	1265 ± 54.32	1835.50 ± 47.15 ^ab^
HCS	848 ± 6.09	1053 ± 44.30	1358 ± 39.78	1923 ± 50.96 ^a^
*p*-value	0.995	0.817	0.229	0.021
**BW gain, g**	40–54	54–68	68–89	40–89
CON	190.50 ± 28.13	207 ± 40.79 ^b^	508.89 ± 47.51	914.44 ± 51.61 ^b^
NCS	170.50 ± 27.88	250.50 ± 19.37 ^ab^	560 ± 34.09	995 ± 56.85 ^ab^
LCS	228.75 ± 15.08	236.50 ± 34.59 ^ab^	570.50 ± 18.32	987.50 ± 59.76 ^ab^
HCS	204.50 ± 19.69	305 ± 20.43 ^a^	565 ± 42.31	1074.50 ± 44.10 ^a^
*p*-value	0.428	0.002	0.653	0.017
**FI, g**	40–54	54–68	68–89	40–89
CON	1172 ± 67.78 ^ab^	1215 ± 67.84	2218.89 ± 26.83 ^b^	4881.67 ± 50.52 ^a^^,^^b^
NCS	1161 ± 15.37 ^ab^	1224.20 ± 27.64	2260.56 ± 14.54 ^ab^	4960.56 ± 17.47 ^a^
LCS	1025.50 ± 23.24 ^b^	1224.50 ± 10.87	2275 ± 0.01 ^a^	4840 ± 14.59 ^b^
HCS	1177 ± 8.80 ^a^	1217.50 ± 27.48	2275 ± 0.01 ^a^	4962.50 ± 27.48 ^a^
*p*-value	<0.001	0.779	0.023	0.018
**FCR**	40–54	54–68	68–89	40–89
CON	6.03 ± 0.47 ^a^	6.23 ± 1.19	4.99 ± 0.81	5.47 ± 0.26 ^a^
NCS	6.52 ± 0.81 ^a^	5.10 ± 0.33	4.27 ± 0.45	5.08 ± 0.23 ^ab^
LCS	4.62 ± 0.33 ^b^	5.57 ± 0.92	4.02 ± 0.12	4.97 ± 0.39 ^ab^
HCS	5.52 ± 0.34 ^ab^	4.14 ± 0.25	4.36 ± 0.51	4.51 ± 0.16 ^b^
*p*-value	0.135	0.291	0.638	0.014

^a,b^ Means in the same column followed by uncommon superscript letters are significantly different (*p* < 0.05). BW = body weight; FI = feed intake; FCR = feed conversion ratio.

## Data Availability

Not applicable.
